# Baseline Susceptibility, Cross-Resistance, and Sublethal Effects of Broflanilide, a Novel Meta-Diamide Pesticide, in *Spodoptera litura*

**DOI:** 10.3390/ijms24065351

**Published:** 2023-03-10

**Authors:** Yunyi Li, Cheng Qu, Qinghe Zhang, Liping Zhang, Chen Luo, Ran Wang

**Affiliations:** 1 Institute of Cotton Research, Shanxi Agricultural University, Yuncheng 044000, China; 2 Institute of Plant Protection, Beijing Academy of Agriculture and Forestry Sciences, Beijing 100097, China

**Keywords:** *Spodoptera litura*, insecticide sensitivity, field-evolved resistance, cross-resistance, sublethal effects, detoxifying enzymes

## Abstract

*Spodoptera litura* is a damaging and notorious insect pest of agricultural crops that has developed resistance to various insecticides. Broflanilide is a novel pesticide with a unique mode of action that displays high efficiency against lepidopterous larvae. We here determined the baseline susceptibility of a laboratory strain of *S*. *litura* to broflanilide and 10 other popular insecticides. Furthermore, we measured susceptibility and cross-resistance using three common insecticides in 11 field-collected *S*. *litura* populations. Broflanilide caused the highest toxicity among all tested insecticides, with the laboratory strain and all field-collected populations showing high susceptibility. Moreover, no cross-resistance was detected between broflanilide and the other tested insecticides. We subsequently evaluated the sublethal effects of broflanilide and found that treatment with the 25% lethal concentration (LC_25_) prolonged the development duration in the larvae, reduced the pupation rate and pupae weight, and decreased egg hatchability. Finally, the activities of three detoxifying enzymes were measured in *S*. *litura* after treatment with the LC_25_ dose. The results suggested that enhanced cytochrome P450 monooxygenase (P450) activity could be involved in broflanilide detoxification. Overall, these findings demonstrate the strong toxicity and significant sublethal effects of broflanilide in *S*. *litura* and suggest that increased P450 activity may be associated with broflanilide detoxification.

## 1. Introduction

Broflanilide is a new meta-diamide pesticide that targets the γ-aminobutyric acid receptor (GABAR) in insect pests through a novel mechanism of action [1]. The Insecticide Resistance Action Committee has classified it in a novel group (group 30) based on its action as an allosteric modulator of GABAR [2]. It not only exhibits excellent lethal effects against a host of lepidopteran pests, such as *Plutella xylostella*, *Helicoverpa armigera*, *Spodoptera frugiperda*, *Spodoptera litura*, and *Spodoptera exigua* [3,4,5], but also shows highly lethal pesticidal activity against other insect pests including thrips, cotton aphids, and two-spotted spider mites [6,7,8]. Broflanilide was registered as a commercialized pesticide in China in 2020 and is considered a promising chemical agent for field application to control agricultural pests [9]. Moreover, it displays minimal non-target effects on various natural crop enemies such as *Cyrtorhinus lividipennis*, *Singa pygmaea*, *Pirata subpiraticus*, *Erigonidium graminicolum*, and *Theridion octomaculatum* [10]. Broflanilide could be effective for controlling herbivores that are resistant to other pesticides, and this compound has broad potential applications in insecticide-resistance management both locally and abroad. Although, on the basis of previous publications and in view of its unique mode of action, broflanilide is considered a very promising chemical agent for controlling pests that are resistant to other insecticides, it is important to study characteristics such as toxicity, baseline field susceptibility, and cross-resistance to establish efficient integrated pest management programs and inform safe usage practices.

Chemical insecticides gradually degrade after field spraying, and the target insect pests are often exposed to low concentrations of them in the field [11,12,13]. Apart from the lethal effects of pesticides, the low residual concentrations can exert sublethal effects, impacting biological, physiological, and biochemical processes, immunological function, development, reproduction of pests and even community ecology [12]. For instance, in *Bemisia tabaci*, sublethal concentrations of various chemical agents (such as afidopyropen, cycloxaprid, cyantraniliprole, clothianidin, and dinotefuran) shorten the duration of insect development and decrease the number of oviposition days, female fecundity, and egg hatchability [14,15,16,17,18]. Similarly, sublethal concentrations of chlorantraniliprole not only extend the duration of larval development and decrease egg hatchability in beet armyworm, but also reduce viability and reproduction in diamondback moth [19]. In contrast, exposure to sublethal pesticide concentrations can stimulate the development and reproduction of some insect pests. These stimulatory effects, referred to as hormesis, occur in many insect pests that are exposed to various pesticides [20,21]. The demonstration of hormesis is critical to management strategies of insect pests, and because hormesis results from sublethal concentrations of pesticides, extensive pesticide application can cause insect pest resurgences [21,22,23].

*Spodoptera litura* (Fabricius), commonly known as tobacco cutworm, is an extensively distributed and notoriously devastating agricultural insect pest. *S. litura* is distributed worldwide in temperate and subtropical zones and attacks hundreds of different crop species [24,25], especially in subtropical and tropical Asian countries such as China, Japan, Pakistan, and India [26,27,28]. Owing to its great capacity of reproduction, over-reliance on insecticides against *S. litura* has contributed to the development of resistance to various insecticides applied around the world, and over the past 10 years in China, excessive reliance on chemical insecticides for crop management has caused *S. litura* to develop significant resistance to different types of pesticides such as carbamates, organophosphates, pyrethroids, and benzoylurea, and novel pesticides such as indoxacarb, metaflumizone, chlorantraniliprole, and pyridalyl [28,29,30,31,32,33]. Continuous over-utilization of these pesticides is unlikely to efficiently control *S. litura*. It is therefore urgent to identify a novel chemical agent for use in rotation with existing pesticides. In the present study, we firstly confirmed the toxicity of broflanilide to *S. litura* and then determined the baseline susceptibility of field-sampled *S. litura* populations and assessed pesticide cross-resistance with other three popular chemical agents. With this work, we found that all field-sampled populations were highly susceptible to broflanilide, and no cross-resistance to the other tested pesticides was observed. Moreover, we assessed the sublethal effects of broflanilide on *S. litura* and then illustrated the biochemical mechanisms associated with these sublethal effects by measuring the activities of esterase (EST), glutathione S-transferase (GST), and cytochrome P450 monooxygenase (P450). In summary, this study describes the optimal use of broflanilide against *S. litura* and lays the foundation for future research and the development of broflanilide as a novel pesticide.

## 2. Results

### 2.1. Toxicity and Baseline Susceptibility of S. litura to Broflanilide

**Figure 1:**
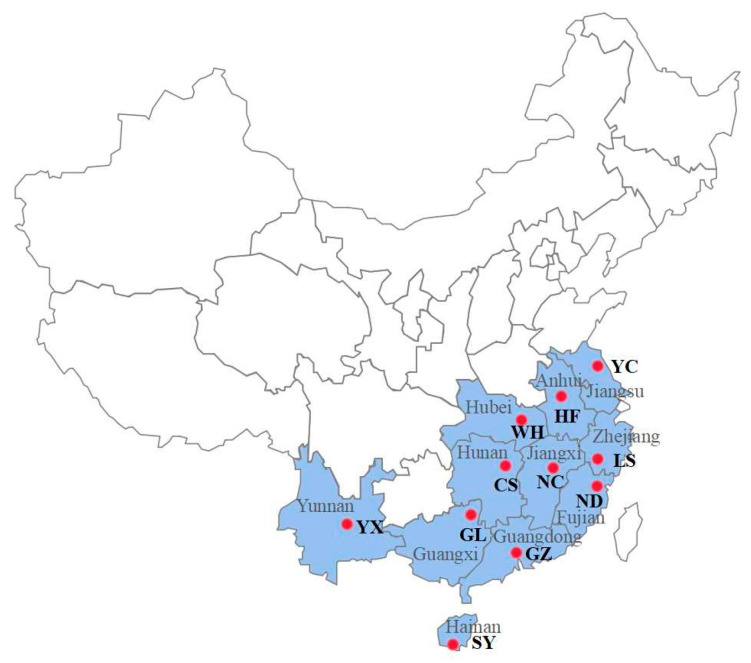
*Spodoptera litura* field populations of Yunnan (Yuxi, YX), Anhui (Hefei, HF), Hubei (Wuhan, WH), Jiangsu (Yancheng, YC), Jiangxi (Nanchang, NC), Zhejiang (Lishui, LS), Fujian (Ningde, ND), Hunan (Changsha, CS), Guangdong (Guangzhou, GZ), Guangxi (Guilin, GL), and Hainan (Sanya, SY) sampling sites in China. Samples were collected in 2021.

**Figure 2:**
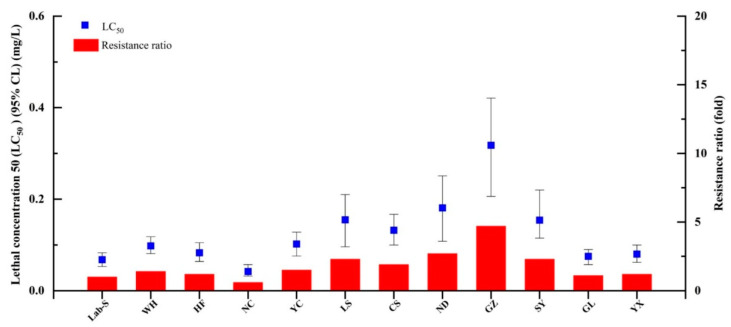
Susceptibility of field-collected *Spodoptera litura* populations to broflanilide. LC_50_, median lethal concentration.

The LC_50_ values were calculated for broflanilide and 10 other popular insecticides using *S. litura* larvae (Table 1). Broflanilide showed the highest toxicity against *S. litura* (LC_50_ = 0.08 mg/L), followed by abamectin (0.10 mg/L), tetraniliprole (0.19 mg/L), spinetoram (0.46 mg/L), chlorfenapyr (0.88 mg/L), chromafenozide (0.91 mg/L), pyridalyl (1.22 mg/L), cyantraniliprole (1.32 mg/L), chlorantraniliprole (2.21 mg/L), metaflumizone (3.61 mg/L), and flubendiamide (9.95 mg/L); these compounds were 1.3, 2.4, 5.8, 11.0, 11.4, 15.3, 16.5, 27.6, 45.1, and 124.4 times less toxic than broflanilide, respectively. The baseline broflanilide susceptibility was then determined in *S. litura* populations collected from 11 Chinese provinces (Figure 1) and compared to that of the susceptible Lab-S strain. Little broflanilide resistance was observed in any of the field populations (Figure 2).

### 2.2. Cross-Resistance to Broflanilide and Three Other Popular Insecticides

Three field-collected populations (GZ, YX, and ND) were used to establish the cross-resistance patterns between broflanilide and three other popular insecticides (metaflumizone, chlorantraniliprole, and pyridalyl) as previously described by our lab [33]. Compared to the reference strain Lab-S, the GZ, YX, and ND populations displayed 80.4-, 64.7-, and 51.8-fold higher resistance, respectively, to metaflumizone; 86.4-, 56.4-, and 59.7-fold higher resistance, respectively, to chlorantraniliprole; and 48.8-, 78.3-, and 40.5-fold higher resistance, respectively, to pyridalyl (Table 2). Compared to the Lab-S strain, the GZ, YX, and ND populations showed 3.3-, 1.8-, 2.1-fold higher resistance, respectively, to broflanilide. Thus, broflanilide displayed little cross-resistance with metaflumizone, chlorantraniliprole, or pyridalyl.

### 2.3. Sublethal Effects of Broflanilide in S. litura

**Figure 3:**
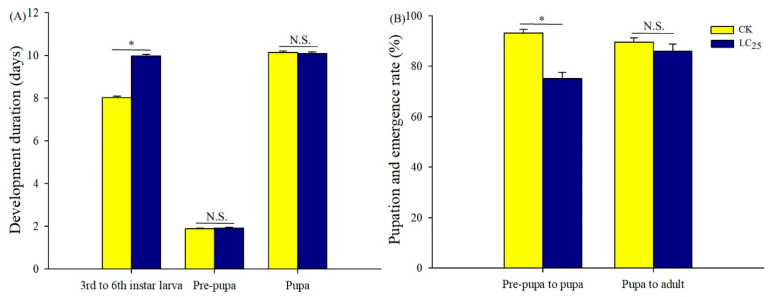
Development duration (**A**) and pupation and emergence rates (**B**) of *Spodoptera litura*. Yellow, control (CK) individuals. Dark blue, individuals treated with the 25% lethal concentration (LC_25_) of broflanilide. Values are presented as the mean ± standard error. * *p* < 0.05 (Student’s *t*-test) and N.S. indicates not significant.

**Figure 4:**
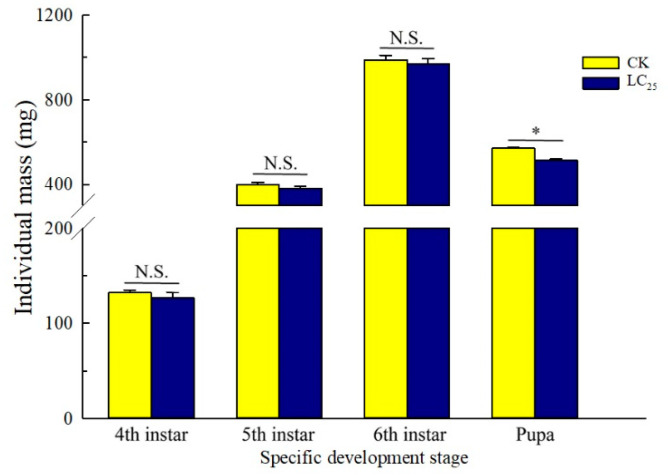
*Spodoptera litura* larval weight at selected developmental stages. Yellow, control (CK) individuals. Dark blue, individuals treated with the 25% lethal concentration (LC_25_) of broflanilide. Values are presented as the mean ± standard error. * *p* < 0.05 (Student’s *t*-test) and N.S. indicates not significant.

**Figure 5:**
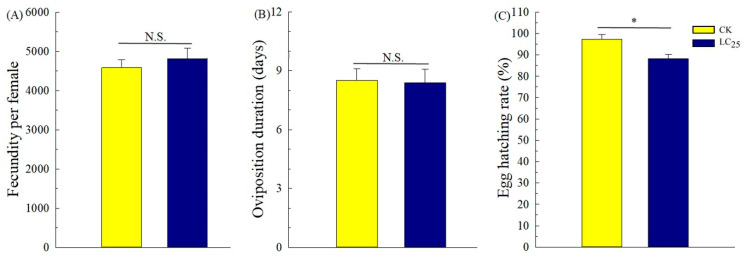
Fecundity (**A**), oviposition duration (**B**), and egg hatching rate (**C**) of *Spodoptera litura*. Yellow, control (CK) individuals. Dark blue, individuals treated with the 25% lethal concentration (LC_25_) of broflanilide. Values are presented as the mean ± standard error. * *p* < 0.05 (Student’s *t*-test) N.S. indicates not significant.

To determine potential sublethal effects of broflanilide, several biological parameters were measured in 3rd-instar larvae treated with the LC_25_ dose: development duration, weight of pupae and larvae, pupation and emergence rate, female fecundity and oviposition duration, and egg hatchability (Figure 3, Figure 4 and Figure 5). In comparison with control individuals, the development duration from 3rd- to 6th-instar larvae was greatly extended (by 1.95 d) in individuals treated with the LC_25_ dose, although the duration of the pre-pupa and pupa stages was not significantly different (Figure 3A). The successful pupation rate was decreased in the LC_25_-treated group compared to the control, yet a small significant difference was observed in the emergence rate (Figure 3B). The mean pupal weight was significantly decreased (by 55.76 mg) in the LC_25_ treatment group compared to the control group, whereas there were no significant differences in weight at any of the other four tested stages (Figure 4). The mean fecundity per female and oviposition duration were not significantly different between the LC_25_ and the control groups, although egg hatchability was significantly reduced (by 9.09%) in the treatment group (Figure 5).

### 2.4. Detoxifying Enzyme Activity in LC_25_-Treated Insects

To confirm the potential functions of *S. litura* detoxifying enzymes in response to sublethal concentrations of broflanilide, P450, GST, and EST activities were assayed in control and LC_25_-treated insects (Table 3). In comparison with the control individuals, those treated with LC_25_ dose showed significantly enhanced P450 activity (1.6-fold higher). Similarly, the LC_25_ treatment group showed significantly increased GST activity, 1.7-fold higher than in the control group. EST activity was little increased in the LC_25_ treatment group compared to the control, but the difference was not significant.

## 3. Discussion

*S. litura* is an economically damaging insect pest that is notorious for its ability to develop pesticide resistance [34]. Due to its notable history of evolving resistance, it is essential to determine *S. litura* baseline susceptibility to novel pesticides before they are applied in the field. We here established the baseline susceptibility of several field-sampled populations of *S. litura* to 10 popular insecticides using our previously published method [33]. This is the first report about the baseline susceptibility of this pest to broflanilide in China. The data of the current research displayed that the novel pesticide broflanilide was greatly effective against *S. litura*. Moreover, we found a narrow range of geographical variation in broflanilide susceptibility between populations (less than five-fold resistance ratio). Another study revealed that field populations of the insect pests *P. xylostella*, *H. armigera*, and *S. frugiperda* in China are highly susceptible to broflanilide [5]. This novel insecticide could thus be a powerful tool to control the four lepidopteran species of the most common and highly damaging insect pests in China. In other orders of agricultural insect pests in China, an increasing number of species have exhibited baseline susceptibility to broflanilide; it has been reported that broflanilide is potentially useful against cotton aphids and several thrip species [7,8]. The utilization of novel pesticides is considered a critical strategy to avoid or delay the development of resistance to common pesticides in agricultural herbivores. Our results, therefore, serve as a valuable reference when monitoring broflanilide resistance in *S. litura*, contributing to the improvement of resistance management measures in China henceforth.

Three field-collected resistant strains of *S. litura* were used to determine the cross-resistance between broflanilide and three other popular insecticides (metaflumizone, chlorantraniliprole, and pyridalyl). These comparisons indicated a little significant cross-resistance, meaning that it is highly feasible to rotate broflanilide with metaflumizone, chlorantraniliprole, and pyridalyl in the field to combat *S. litura*. Similarly, significant cross-resistance to broflanilide was not observed in three diamide-resistant populations of diamondback moth and one spinosyns-resistant population of fall armyworm [5]. Earlier reports indicated that broflanilide displays excellent efficiency against fipronil- and dieldrin-resistant housefly, fipronil-resistant *Sogatella furcifera* and *Oulema oryzae*, diamide-resistant diamondback moth [1,35], and dieldrin- and pyrethroid-resistant *Anopheles gambiae* [36,37]. All things considered, our results suggest that there is minimal or no cross-resistance between broflanilide and other common pesticides that are associated with diverse mechanisms of resistance. Broflanilide can thus be a helpful tool to complete the management of pests that are already resistant to popular chemical agents.

In addition to killing insects at lethal concentrations, sublethal concentrations of chemical agents can exert significant effects on insect behavior, physiology, and even community ecology. These effects must be studied as part of an integrated evaluation of pesticide effects [12]. We here found that the LC_25_ dose of broflanilide greatly slowed larval development, decreased the pupation rate and pupae weight, and reduced egg hatchability in *S. litura*. Other studies previously showed that a variety of chemical agents exert sublethal effects on *S. litura*, interfering with its development and reproduction [38,39,40]. A recent study of broflanilide effects on *S. frugiperda* showed that sublethal doses were associated with decreased larval body length, prolonged larval and pupal duration, and malformed development of pupae and adults [41]. In *Tetranychus urticae*, sublethal concentrations of broflanilide not only reduced the total insect’s life span, but decreased the fecundity of adult females, causing a population decline [6]. Although hormesis has been reported in several insect species as a result of exposure to various insecticides [21], such effects have not been reported for broflanilide. Furthermore, we found that the activities of GST and P450, two major detoxifying enzymes in insects, were significantly increased in *S. litura* after exposure to the LC_25_ dose of broflanilide. In *B. tabaci*, treatment with the LC_25_ dose of β-asarone (a plant-derived potential insecticide) significantly induces P450 activity, and a sublethal concentration of afidopyropen enhances GST activity [18,42]; in contrast, GST activity is significantly inhibited in *Panonychus citri* treated with sublethal concentrations of the acaricides fenazaquin and acequinocyl [43]. Based on the RNA-seq technology, recent studies have suggested that the mechanisms of insecticide sublethal effects are strongly associated with detoxifying gene expression, and those results indicate that cytochrome P450 monooxygenases, esterases, glutathione S-transferases, and ATP-binding cassette transporters could be up- or down-regulated with exposure to sublethal concentrations of insecticides [44,45]. Transcriptomic analyses will therefore be carried out in *S. litura* treated with sublethal doses of broflanilide to identify related transcriptional changes, understand the functions of detoxifying genes, and finally delineate the mechanisms of action of this insecticide.

## 4. Materials and Methods

### 4.1. Insects

The lab-raised susceptible *S. litura* strain Lab-S was reared as previously described [33] with no pesticide exposure for over five years. Eleven field populations of *S. litura* were collected from southern China (Figure 1) and named Yunnan (Yuxi, YX), Anhui (Hefei, HF), Hubei (Wuhan, WH), Jiangsu (Yancheng, YC), Jiangxi (Nanchang, NC), Zhejiang (Lishui, LS), Fujian (Ningde, ND), Hunan (Changsha, CS), Guangdong (Guangzhou, GZ), Guangxi (Guilin, GL), and Hainan (Sanya, SY). Among the above field-collected populations of *S. litura*, the GZ, YX, and ND populations displayed middle to high levels of resistance to the three insecticides metaflumizone, chlorantraniliprole, and pyridalyl, respectively, according to our previous work [33]. The GZ, YX, and ND populations and the Lab-S strain were used to establish the cross-resistance patterns. All populations were maintained in a well-controlled growth chamber at 26 ± 2 °C with 65 ± 5% relative humidity and a 16/8 h light/dark photoperiod. All larval populations were fed an artificial diet, and adults were reared on a 10% sugar solution.

### 4.2. Insecticides and Chemicals

The insecticides and chemicals utilized for this study were analytical-grade standards. Broflanilide (Chemical Abstracts Service [CAS] #1207727-04-5), tetraniliprole (CAS #1229654-66-3), chlorantraniliprole (CAS #500008-45-7), chromafenozide (CAS #143807-66-3), and spinetoram (CAS #187166-40-1) were purchased from Dr. Ehrenstorfer (Augsburg, Germany). Cyantraniliprole (CAS #736994-63-1), flubendiamide (CAS #272451-65-7), pyridalyl (CAS #179101-81-6), metaflumizone (CAS #139968-49-3), chlorfenapyr (CAS #122453-73-0), abamectin (CAS #71751-41-2), dimethyl sulfoxide (DMSO) (CAS #67-68-5), and Triton X-100 (CAS #9002-93-1) were purchased from Sigma Aldrich (Shanghai, China).

### 4.3. Bioassays

All bioassays in this study were carried out with the use of a previously published leaf-dip method [33] with slight changes. Third-instar larvae were randomly sampled, and working concentrations of the pesticides to be tested were generated by dilution in DMSO and sterile water with 0.1% Triton X-100. Leaf discs (4.5 cm in diameter) were cut from *Brassica oleracea* (cabbage), dipped into a working concentration of pesticide for 20 s, dried at room temperature in the growth chamber, then put into a Petri dish (5 cm in diameter). Ten 3rd-instar larvae were placed onto each leaf disc to form one replication. There were four replicates for each working concentration of each pesticide. All larvae were maintained in a well-controlled growth chamber under the conditions described above.

### 4.4. Evaluation of Sublethal Broflanilide Effects on S. litura

To assess the sublethal effects of broflanilide on *S. litura*, leaf discs were prepared with the 25% lethal concentration (LC_25_) of broflanilide (0.03 mg/L) using the leaf-dip method described above. The leaf discs were then incubated with 150 12-h-old 3rd-instar larvae for 48 h to generate the LC_25_ treatment group. The control group comprised an additional 150 untreated third-instar larvae. The larvae in each treatment group were randomly divided into 15 biological replicate groups containing 10 larvae each. After pupation, the deformed pupae were counted, and the pupation rate was recorded. After the adults emerged, the rate of emergence, male/female ratio, and deformed adult rate were recorded. Fifteen pairs of female and male adults were coupled within 12 h and put in a plastic cup (4 × 8 cm in diameter × height) containing a 10% (*w*/*v*) honey solution, which was replaced daily. Longevity was measured daily for male and female adults; for female adults, the duration of oviposition and the number of eggs were also recorded every day.

### 4.5. Detoxifying Enzyme Assays in LC_25_-Treated Insects

Fifteen 3rd-instar larvae were selected and homogenized in 20 mL of homogenization buffer (0.1 M phosphate buffer at pH 7.6 with 1 mM EDTA, 1 mM PTU, 1 mM DTT, 20% glycerol, and 1 mM PMSF). The samples were centrifuged at 4 °C and 12,000× *g* for 20 min. The supernatant was removed and transferred to a new Eppendorf tube on ice, then immediately assayed for protein content and P450, EST, and GST activity using a previously published method [46] with slight changes. P450 activity was determined using p-nitroanisole as the substrate; for the p-nitroanisole O-demethylation (PNOD) assay, the activity was measured in nmol p-nitrophenol min^−1^ mg^−1^ protein. GST activity was measured using 1-chloro-2, 4-dinitrobenzene (CDNB) as the substrate and computed using an extinction coefficient of 9.6 mM^−1^ cm^−1^ for CDNB [47]. EST activity was assayed using α-naphthyl acetate (α-NA) as the substrate and measured in nmol α-naphthol min^−1^ mg^−1^ protein. The total protein content was measured using bovine serum albumin (BSA) as the standard, as described by Bradford [48]. There were three replicates per treatment group for each assay.

### 4.6. Statistical Analysis

Probit analysis was conducted to confirm the significance of the death rate statistics in the samples treated with a series of working concentrations of chemical agents. The concentration–mortality response, median lethal concentration (LC_50_), 95% fiducial limit (FL), and slope value were calculated for each compound with PoloPlus [49]. The resistance ratio (RR) was estimated as LC_50_ (field-collected population)/LC_50_ (Lab-S), and the levels of insecticide resistance are published by our previous work [33]. Specifically, susceptibility corresponded to the RR less than 5-fold higher than the reference value, low level of resistance corresponded to the RR from 5- to 10-fold higher, middle level of resistance corresponded to the RR from 10- to 40-fold higher, high level of resistance corresponded to the RR from 40- to 160-fold higher, and very high level of resistance corresponded to the RR over 160-fold higher. Student’s *t*-test was performed to determine the statistical significance of the differences in growth duration, viability, fecundity, oviposition time, and egg hatchability of *S. litura* between the LC_25_-treated and the control groups. Student’s *t*-test was also used to assess differences in detoxifying enzyme activity between the LC_25_-treated and the control groups. All statistical analyses were conducted in SPSS [50].

## 5. Conclusions

In the current work, firstly, we found that the novel meta-diamide pesticide, broflanilide, is the most toxic to larvae of *S. litura* among eleven popular commercialized chemical agents which are commonly used against *S. litura*. After that, we monitored the status of resistance to broflanilide by using eleven populations of *S. litura* field-collected across southern China and established for the first time the baseline susceptibility to broflanilide of *S. litura* in China. We showed that the susceptibility was very high, and no significant resistance was detected in China. After that, the cross-resistance patterns with the three common insecticides metaflumizone, chlorantraniliprole, and pyridalyl were established using three field-evolved resistant populations of *S. litura*, and no cross-resistance between broflanilide and the three tested insecticides was observed. Then, the sublethal effects of broflanilide were evaluated, and after treatment with the 25% lethal concentration (LC_25_) of the third-instar larvae, we found the development duration of the larvae was prolonged, the pupation rate and pupae weight were reduced, and egg hatchability was decreased. Based on the LC_25_ treatment, the activities of the three main detoxifying enzymes cytochrome P450 monooxygenase (P450), glutathione S-transferase (GST), and esterase (EST) were estimated in S. *litura* after the treatment, and the results indicated that increased P450 activity could contribute to the detoxification of broflanilide. Overall, all the above findings illustrated the high toxicity and significant sublethal effects of broflanilide in *S. litura* and showed that increased P450 activity could be related ti the detoxification of broflanilide.

## Figures and Tables

**Table 1:** Toxicitiy of broflanilide and 10 other popular insecticides in the susceptible *Spodoptera litura* strain Lab-S.

Insecticide	N ^a^	LC_50_ (95% CL) (mg L^−1^) ^b^	Slope ± SE	*X*^2^ (*df*)
Broflanilide	200	0.08 (0.06–0.10)	2.09 ± 0.31	1.16 (3)
Abamectin	200	0.10 (0.08–0.13)	1.68 ± 0.25	2.50 (3)
Tetraniliprole	200	0.19 (0.15–0.24)	1.85 ± 0.13	2.10 (3)
Spinetoram	200	0.46 (0.31–0.60)	1.69 ± 0.26	1.28 (3)
Chlorfenapyr	200	0.88 (0.69–1.12)	1.89 ± 0.26	1.15 (3)
Chromafenozide	200	0.91 (0.73–1.12)	2.22 ± 0.28	1.60 (3)
Pyridalyl	200	1.22 (0.93–1.54)	1.97 ± 0.27	1.24 (3)
Cyantraniliprole	200	1.32 (0.96–1.72)	1.67 ± 0.25	0.96 (3)
Chlorantraniliprole	200	2.21 (1.57–3.21)	1.26 ± 0.25	0.91 (3)
Metaflumizone	200	3.61 (2.52–4.79)	1.55 ± 0.25	0.81 (3)
Flubendiamide	200	9.95 (8.37–11.82)	1.43 ± 0.13	2.53 (3)

^a^ Number of insects used. ^b^ CL, confidence limit.

**Table 2:** Cross-resistance between broflanilide and three popular insecticides in *Spodoptera litura*.

Insecticide	Strain	N ^a^	LC_50_ (95% CL) (mg/L) ^b^	Slope ± SE	*χ*^2^ (*df*)	RR ^c^
Broflanilide	Lab-S	200	0.06 (0.05–0.07)	2.56 ± 0. 33	2.03 (3)	
	GZ	200	0.20 (0.16–0.26)	1.76 ± 0.25	2.62 (3)	3.3
	YX	200	0.11 (0.08–0.14)	1.70 ± 0.26	2.34 (3)	1.8
	ND	200	0.13 (0.11–0.15)	1.71 ± 0.15	1.45 (3)	2.1
Metaflumizone	Lab-S	200	4.64 (3.17–6.21)	1.51 ± 0.25	2.80 (3)	
	GZ	200	373.21 (300.98–460.38)	2.27 ± 0.28	1.62 (3)	80.4
	YX	200	300.04 (239.71–365.52)	2.49 ± 0.31	1.22 (3)	64.7
	ND	200	240.18 (184.11–311.72)	1.74 ± 0.25	1.57 (3)	51.8
Chlorantraniliprole	Lab-S	200	3.36 (2.59–4.29)	1.84 ± 0.25	2.30 (3)	
	GZ	200	290.38 (221.52–400.66)	1.62 ± 0.24	1.45 (3)	86.4
	YX	200	189.45 (146.00–245.35)	1.77 ± 0.25	2.97 (3)	56.4
	ND	200	200.51 (150.78–256.41)	1.83 ± 0.26	1.14 (3)	59.7
Pyridalyl	Lab-S	200	1.18 (0.88–1.51)	1.87 ± 0.17	1.77 (3)	
	GZ	200	57.56 (41.06–75.21)	1.68 ± 0.25	2.05 (3)	48.8
	YX	200	92.37 (64.91–120.09)	1.82 ± 0.27	1.22 (3)	78.3
	ND	200	47.81 (35.11–64.60)	1.48 ± 0.24	1.01 (3)	40.5

^a^ Number of insects used. ^b^ CL, confidence limit. ^c^ Resistance ratio (RR) = median lethal concentration (LC_50_) of resistant strain/LC_50_ of Lab-S.

**Table 3:** Detoxification enzyme activities in *Spodoptera litura* in the control (CK) and 25% lethal concentration (LC_25_) broflanilide treatment groups ^a^.

Treatment	P450 Activity		EST Activity		GST Activity	
	nmol min^−1^ mg^−1^	Ratio ^b^	nmol min^−1^ mg^−1^	Ratio ^b^	nmol min^−1^ mg^−1^	Ratio ^b^
CK	0.031 ± 0.003		115.71 ± 4.9		733.4 ± 20.1	
LC_25_	0.050 ± 0.004 *	1.6	130.14 ± 6.2	1.1	1255.3 ± 35.8 *	1.7

^a^ Mean activity values in a single column followed by asterisks are significantly different at *p* < 0.05 (Student’s *t*-test). ^b^ Ratio = activity in the LC_25_ treatment group/activity in the CK group.

## Data Availability

All relevant data are available from the corresponding author on request (wangran@ipepbaafs.cn).
